# Decreased Endometrial IL-10 Impairs Endometrial Receptivity by Downregulating HOXA10 Expression in Women with Adenomyosis

**DOI:** 10.1155/2018/2549789

**Published:** 2018-12-31

**Authors:** Junxia Wang, Chenyang Huang, Ruiwei Jiang, Yali Du, Jianjun Zhou, Yue Jiang, Qiang Yan, Jun Xing, Xiaoni Hou, Jidong Zhou, Haixiang Sun, Guijun Yan

**Affiliations:** ^1^Reproductive Medicine Center, Drum Tower Clinic Medical College of Nanjing Medical University, Nanjing 210008, China; ^2^Reproductive Medicine Center, Department of Obstetrics and Gynecology, Affiliated Drum Tower Hospital of Nanjing University Medical School, Nanjing 210008, China; ^3^Department of Obstetrics and Gynecology, Wuxi People's Hospital Affiliated with Nanjing Medical University, Wuxi 214002, China

## Abstract

**Objective:**

The aim of this study was to investigate the potential role of IL-10 in regulating the receptivity marker HOXA10 in the endometrium of women with adenomyosis.

**Methods:**

The expression levels of IL-10, HOXA-10, STAT3, and p-STAT3 in the endometrium of women with adenomyosis and controls were examined by means of western blotting and immunohistochemistry. The expression of the HOXA10 protein in Ishikawa cells treated with rIL-10 was examined by western blotting. The attachment rate of BeWo cell spheroids to Ishikawa cells treated with rIL-10 was expressed as a percentage of the total number of spheroids.

**Results:**

The expression levels of HOXA10 and IL-10 in the adenomyosis group were significantly lower than those in the control group, and there was a positive correlation between HOXA10 and IL-10 protein levels in all the women examined. rIL-10 increased HOXA10 expression in a concentration- and time-dependent manner by inducing the phosphorylation of STAT3 in Ishikawa cells. Treatment with rIL-10 promoted the attachment of BeWo spheroids to Ishikawa cells, which was reversed by the inhibition of STAT3 phosphorylation. The expression of p-STAT3 in the adenomyosis group was significantly lower than that in the control group, and there was a positive correlation between IL-10 and p-STAT3 protein levels in all the women examined.

**Conclusions:**

Both IL-10 and HOXA10 levels in the endometrium are significantly reduced in women with adenomyosis compared with those in control women. The phosphorylation of STAT3 has been proven to be a critical mediator between IL-10 and HOXA10, which may play critical roles in embryo implantation.

## 1. Introduction

Uterine adenomyosis is characterized as the benign invasion of ectopic endometrium into the myometrium, with reactive hyperplasia of the surrounding smooth muscle myometrial cells [1]. Studies have revealed that women with adenomyosis experience a 28% reduction in the likelihood of clinical pregnancy after in vitro fertilization (IVF)/intracytoplasmic sperm injection (ICSI) and an increased risk of early pregnancy loss compared to that of normal women [2]. However, the exact mechanism of adenomyosis-associated embryo implantation failure needs to be clarified.

Successful implantation of high-quality embryos requires uterine transition into the receptive state, which is adjusted by sex steroids, growth factors, cytokines, and adhesion molecules [3–5]. Abnormal expression levels of several implantation-related factors (such as HOXA10, LIF, MMP2, interleukin-6, cytochrome P450, and RCAS1) in the eutopic endometrium of women with adenomyosis have been found to result in impaired embryo implantation [6–9]. Among these factors, HOXA10 has emerged as an important factor in endometrial receptivity that is indispensable for embryo adhesion [10–12]. HOXA10 is a homeobox-containing transcription factor sharing a highly conserved homeodomain structurally related to the helix-turn-helix motif of prokaryotic DNA-binding proteins that exhibit sequence-specific DNA-binding activity. Altered expression of endometrial HOXA10, which is relevant to increased estrogen and progesterone levels, has been demonstrated to peak in the midsecretory phase, corresponding to the window of embryo implantation [13–15]. Mice with targeted disruption of* Hoxa10* experience implantation failure because of defective endometrial receptivity [16].* HOXA10* gene expression is decreased in the secretory-phase endometrium of women with adenomyosis [7]. However, the exact mechanisms responsible for the decreased expression of HOXA10 in the human endometrium are unclear.

According to the important effects of cytokines on endometrial conditions for embryo implantation, accumulating evidence indicates that adenomyosis alters endometrial cytokine production, which is suggestive of damage to endometrial receptivity [17, 18]. Interleukin-10 (IL-10) is a critical immunomodulatory cytokine that has been described as a cytokine synthesis inhibitory factor for T lymphocytes produced by T helper 2 (Th2) cell clones and has been shown to inhibit interferon-*γ* synthesis in Th1 cell clones [19]. Numerous studies suggest that IL-10 is one of the major anti-inflammatory cytokines and contributes to the establishment and maintenance of immunosuppression [20], which is speculated to be necessary for endometrial receptivity. An investigation of endometrial cytokine profiles indicated that IL-10 expression is lower in endometrium secretions from women with adenomyosis than in normal controls during the implantation window, which may correlate with compromised endometrium receptivity [21]. Another investigation found that the eutopic endometrium of women with adenomyosis exhibited a higher IL-10 staining intensity than that of normal controls [22]. Thus, the expression level of IL-10 in the endometrium of women with adenomyosis is unclear, and whether IL-10 is involved in the regulation of endometrial receptivity or embryo implantation needs to be further investigated.

In this study, we aimed to characterize the molecular changes in IL-10 and HOXA10 in the endometrium in relation to subfertility in women with adenomyosis and to explore the potential regulatory relationship between these characteristics. In addition, an in vitro blastocyst-like spheroid implantation model was used to evaluate the role of IL-10 in endometrial receptivity. Our study provides a novel molecular mechanism to be considered in relation to the detrimental effect of adenomyosis on reproductive outcomes.

## 2. Materials and Methods

### 2.1. Patients and Sample Collection

Endometrial biopsies for this study were obtained from women attending the Center for Reproductive Medicine of Nanjing Drum Tower Hospital. Endometrial samples were collected during the midsecretory phase (LH+ 7 of the menstrual cycle) using an endometrial curette. Secretory endometria were obtained from 23 women with adenomyosis and 23 controls. Among these samples, 20 samples from the adenomyosis and control groups were stored at −80°C for protein extraction for western blot analysis, and 3 samples from both groups were embedded in paraffin for immunohistochemical analysis. The details of these patients are summarized in Table 1.

All of the women had good hormonal reserves (follicle-stimulating hormone (FSH) on day 3 of the cycle < 10 mIU/mL). None of the patients had received hormonal therapy during the 3 months prior to surgery. The diagnostic criteria for adenomyosis included (1) clinical symptoms: secondary and progressive dysmenorrhea, menorrhagia, and menostaxis; (2) clinical signs: homogeneous enlargement or local uplift of the uterus, firmness, and tenderness; (3) more than two of the following five sonographic features: (1) no distinction of the endometrial-myometrial junction, (2) asymmetry of the anterior and posterior myometrium, (3) subendometrial myometrial striations, (4) myometrial cysts and fibrosis, and (5) heterogeneous myometrial echo texture, as revealed by vaginal ultrasound examination [23]. All patients underwent transvaginal ultrasound performed by 2 investigators experienced in gynecologic imaging. The diagnosis of adenomyosis was established by unifying the clinical symptoms, clinical signs, and ultrasonogram. The control group was composed of women with tubal factor infertility. The inclusion criteria were as follows: indication for IVF; age 40 years or younger; no hydrosalpinx; no polycystic ovarian syndrome (PCOS); no previous surgery for adenomyosis; no uterine malformations; no abnormal uterine bleeding; and no endometrial abnormalities, as assessed by transvaginal ultrasound.

All samples were collected with the informed consent of the patients. Independent ethical approval was obtained from the Nanjing Drum Tower Hospital Research Ethics Committee (no. 201616501).

### 2.2. Cell Culture and Treatment

Ishikawa (a well-differentiated human endometrial adenocarcinoma cell line) and BeWo (human choriocarcinoma cell line) cells were maintained in DMEM/F12 and RPMI culture media, respectively, supplemented with 10% fetal bovine serum (Gibco, BRL/Invitrogen, Carlsbad, CA, USA) and 1% penicillin/streptomycin (HyClone, South Logan, UT, USA). After serum starvation for 12 h, Ishikawa cells were treated with different concentrations (0-100 ng/ml) of rIL-10 (Peprotech, Burlington, NC, USA) for different times (0.5-48 h). To inhibit the STAT3-dependent signaling pathway, Ishikawa cells were treated with 4.6 *μ*M cryptotanshinone (Sigma, St. Louis, MO, USA) for 24 h before rIL-10 treatment.

### 2.3. RNA Isolation and Quantitative Real-Time PCR (qRT-PCR)

Total RNA was extracted from Ishikawa cells using the TRIzol reagent (Invitrogen, Carlsbad, CA, USA), according to the manufacturer's instructions. One microgram of total RNA was reverse transcribed in a total volume of 20 *μ*l. Reverse transcription was performed using random primers, and qRT-PCR was conducted with a MyiQ Single-Color Real-Time PCR Detection System (Bio-Rad, Hercules, CA, USA). The following primers were used for the indicated genes: HOXA10, 5′-GCCCCTTCCGAGAGCAGCAAAG-3′ and 5′-AGGTGGACGCTGCGGCTAATCTCTA-3′; 18S rRNA, 5′-CGGCTACCACATCCAAGGAA-3′ and 5′-CTGGAATTACCGCGGCT-3′. Reactions were run in duplicate using RNA samples from three independent experiments. The fold change in the expression of each gene was calculated via the 2-ΔΔ CT method, with 18S rRNA as an internal control.

### 2.4. Western Blot Analysis

Tissues and cells were homogenized in whole-cell lysis buffer (50 mM Tris-HCl [pH 7.6], 150 mM NaCl and 1.0% NP-40) containing phosphatase and protease inhibitors. Immunoblotting was performed with primary antibodies against HOXA10 (1:1000; Santa Cruz Biotechnology, Santa Cruz, CA, USA), IL-10 (1:1000; Bioworld, St. Louis Park, MN, USA), STAT3 (1:1000; Cell Signaling Technology, Danvers, MA, USA), p(Y705)-STAT3 (1:1000; Bioworld), or GAPDH (1:10,000; Bioworld), followed by a donkey anti-goat or goat anti-rabbit secondary antibody conjugated with horseradish peroxidase (HRP). Detection was performed using an enhanced chemiluminescence kit (Millipore, Billerica, MA, USA).

### 2.5. Immunohistochemistry

Endometrial tissues were fixed in 10% neutral-buffered formalin for 24 h, routinely processed and embedded in paraffin. Tissue sections were immunostained with primary antibodies against HOXA10 (1:50; Abcam, Cambridge, CA, USA), IL-10 (1:1000; Abcam), STAT3 (1:1000; Abcam), or p(Y705)-STAT3 (1:1000; Bioworld) overnight at 4°C, followed by incubation with rabbit anti-goat IgG or goat anti-rabbit IgG and an avidin-biotin complex (Boster Biological Technology, Wuhan, Hunan, China) for 1 h each at room temperature. Finally, the sections were stained with 3,3-diaminobenzidine (DAB) and counterstained with hematoxylin. Control sections were run concurrently with the experimental sections using nonspecific goat IgG and rabbit IgG, and they were similarly pretreated. Nonspecific staining was not detected in the controls. Quantitative analysis was performed using the Image Pro Plus System 6.0 (Media Cybernetics, Inc., Silver Spring, MD, USA) in a blinded fashion, without knowledge of the tissue source. The representative objective protein staining intensity (indicating the relative expression level) was determined according to the mean and integrated optical density (IOD) of the digital image (×400) according to the software's instructions. Signal density data for the tissue areas were obtained from five randomly selected fields of view and subjected to statistical analysis.

### 2.6. Blastocyst-Like Spheroid (BLS) Attachment Model

As the BLS attachment model has been demonstrated to be an accurate and effective in vitro assay for use in endometrial receptivity research, it was employed in this study with some modification [24, 25]. Briefly, a single-cell suspension of BeWo cells was transferred to a Petri dish coated with the antiadhesive polymer poly-2-hydroxyethyl methacrylate (Sigma) to induce the formation of BeWo spheroids that were 150–200 *μ*m in diameter. Simultaneously, a confluent monolayer of Ishikawa cells was infected with 100 ng/mL rIL-10 in a 24-well culture plate for 12 h or 24 h. Fifty spheroids were transferred per chamber onto the confluent monolayer of Ishikawa cells. After incubation of the spheroids for 1.5 h, the attached spheroids were counted, and the attachment rate was expressed as a percentage of the total number of spheroids (% adhesion). All cocultures were monitored using a microscope (Leica, Wetzlar, Germany).

### 2.7. Statistical Analysis

Each experiment was repeated at least three times. All values are expressed as the mean ± SEM or the median and interquartile range, as specified. Student's t-test was used for comparisons of two groups, and ANOVA was applied for experiments involving more than two groups. Correlations between two variables were determined using the Spearman rank correlation coefficient. A* P* value < 0.05 was considered statistically significant.

## 3. Results

### 3.1. Reduced Expression of HOXA10 and IL-10 in Women with Adenomyosis

**Figure 1:**
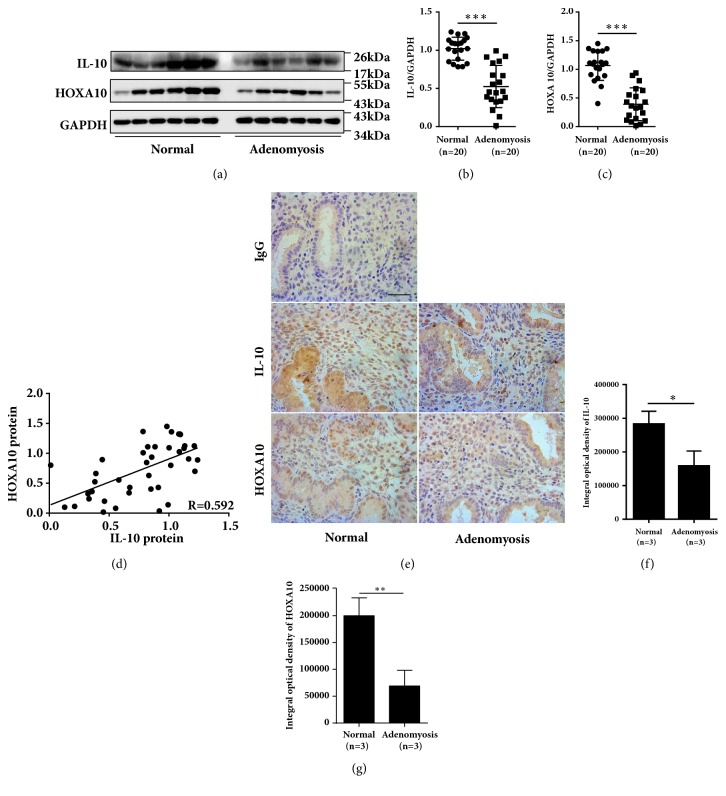
Reduced expression of HOXA10 and IL-10 in women with adenomyosis. Timed midsecretory endometrial biopsies from normal control women (n=20) and women with adenomyosis (n=20) were analyzed for IL-10 and HOXA10 expression via western blotting (a), and the expression levels of IL-10 (b) and HOXA10 (c) were compared between the two groups. The correlation between IL-10 and HOXA10 protein levels was analyzed in all of the women (n=40) (d). Representative images of IL-10 and HOXA10 staining in endometria from women with adenomyosis and normal controls are presented (e). The integrated optical densities (IODs) of total IL-10 (f) and total HOXA10 (g) were compared between the two groups (n=3).The data are plotted as the mean ± SEM. *∗*P<0.05; *∗∗*P<0.01; *∗∗∗*P<0.001.

To assess the mechanisms by which adenomyosis affects implantation, we detected the expression of the endometrial receptivity marker protein HOXA10 and the anti-inflammation cytokine IL-10 in women with adenomyosis. The endometrial HOXA10 protein level was severely decreased (by approximately 50%) in women with adenomyosis compared with that in normal controls (Figures 1(a) and 1(c), P < 0.001). IL-10 expression was reduced by 40% in the endometrium of women with adenomyosis compared with that in normal controls (Figures 1(a) and 1(b), P < 0.001). Interestingly, there was a positive correlation between the protein levels of HOXA10 and IL-10 (Figure 1(d), r = 0.592, P < 0.01). Furthermore, the cellular localization of HOXA10 and IL-10 in endometrium cells was examined by means of immunohistochemistry staining. As shown in Figure 1(e), in normal endometrial cells from controls, the HOXA10 and IL-10 proteins were localized in both epithelial and stromal cells. Integrated optical density (IOD) measurements of the HOXA10 (P < 0.01) and IL-10 (P < 0.05) proteins were significantly lower in women with adenomyosis than in normal controls (Figures 1(f) and 1(g)).

### 3.2. IL-10 Increases HOXA10 Expression via Phosphorylation of STAT3

**Figure 2:**
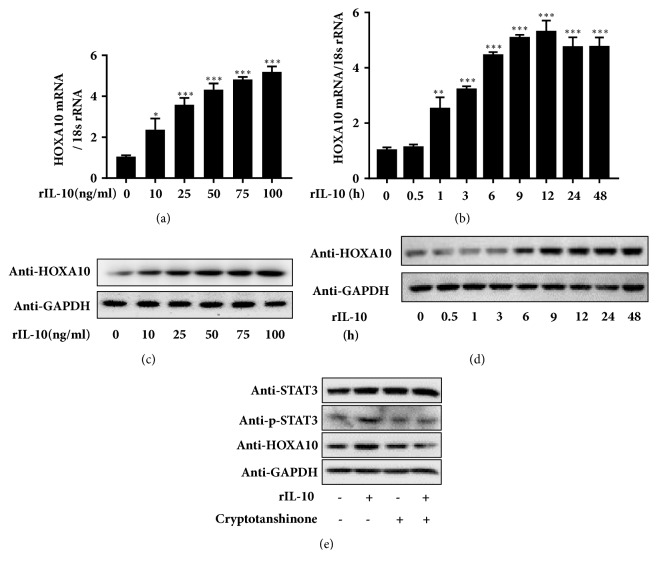
IL-10 increases HOXA10 expression via phosphorylation of STAT3. The expression of HOXA10 mRNA and protein was examined by qRT-PCR and western blotting in Ishikawa cells treated with different concentrations of rIL-10 for 12 h ((a) and (c)) or with 100 ng/mL rIL-10 for different times ((b) and (d)). The expression levels of STAT3, p-STAT3, and HOXA10 were examined by western blotting in Ishikawa cells treated with 4.6 *μ*M cryptotanshinone for 24 h, followed by 100 ng/mL rIL-10 for 12 h, or in controls (e).

HOXA10 expression increased at both the mRNA and protein levels in Ishikawa cells after 12 h of stimulation with rIL-10 in a concentration-dependent manner and was up to 5-fold higher in response to treatment with 100 ng/mL rIL-10 (Figures 2(a) and 2(c)). The treatment of Ishikawa cells with 100 ng/mL rIL-10 induced HOXA10 mRNA and protein expression in a time-dependent manner, resulting in maximal expression at the 12 h point, persisting for up to 24 h after treatment, as determined by qRT-PCR and western blot analyses, respectively (Figures 2(b) and 2(d)). The phosphorylation of STAT3 was suppressed by treatment with 4.6 *μ*M cryptotanshinone, which specifically prevented the increase in HOXA10 expression after treatment with rIL-10 in Ishikawa cells, indicating that the phosphorylation of STAT3 participates in the positive regulation of HOXA10 expression by IL-10 (Figure 2(e)).

### 3.3. IL-10 Promotes BeWo Spheroid Attachment to Ishikawa Cells

**Figure 3:**
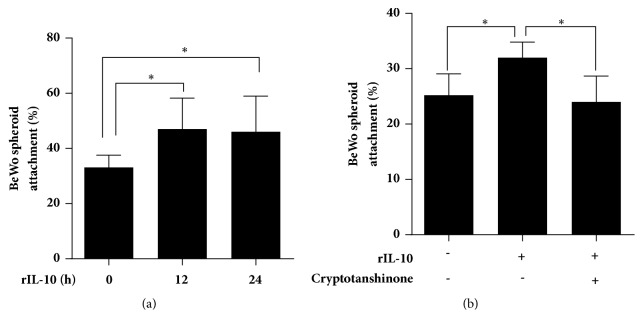
IL-10 promotes BeWo spheroid attachment to Ishikawa cells. Adhesion experiments with BeWo spheroids attached to the Ishikawa cell monolayer. The Ishikawa cells were treated with 100 ng/mL rIL-10 for 12 h or 24 h (a) or with 4.6 *μ*M cryptotanshinone for 24 h, followed by 100 ng/mL rIL-10 for 12 h (b). The presented data are the average of three independent experiments (n=3). The data are plotted as the mean ± SEM. *∗*P<0.05.

As shown in Figure 3(a), treatment of Ishikawa cells with rIL-10 increased the rate of BeWo spheroid attachment compared with that observed in the control group at both the 12 h point (33.1±4.49% vs. 47.0±11.25%, P < 0.05) and 24 h point (33.1±4.49% vs. 46.0±12.99%, P < 0.05), but no difference was found between the 12 h and 24 h points. However, cryptotanshinone-mediated inhibition of STAT3 phosphorylation in Ishikawa cells reversed the facilitating effect of rIL-10 on BeWo spheroid attachment (rIL-10 vs. rIL-10 + cryptotanshinone = 44.0±7.62% vs. 30.0±7.87%, respectively, P < 0.05, Figure 3(b)). These data suggest that IL-10 contributes to embryo attachment via activating the phosphorylation of STAT3 in vitro.

### 3.4. Impaired Phosphorylation of STAT3 in Endometria from Women with Adenomyosis

**Figure 4:**
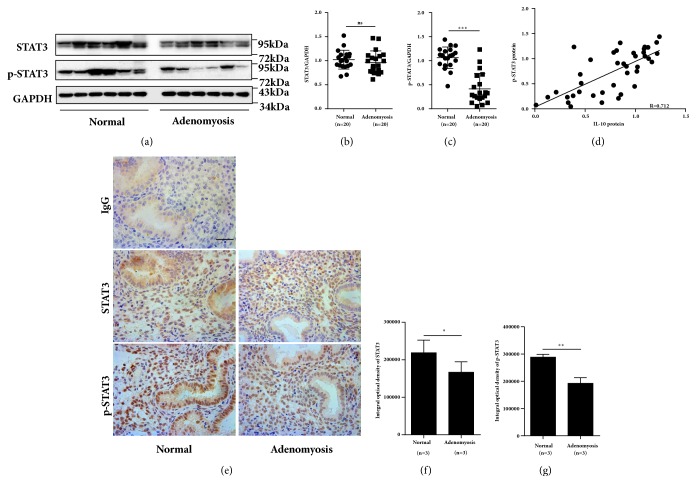
Impaired phosphorylation of STAT3 in endometria from women with adenomyosis. Timed midsecretory endometrial biopsies from normal control women (n=20) and women with adenomyosis (n=20) were analyzed for STAT3 and p-STAT3 expression via western blotting (a), and the expression levels of STAT3 (b) and p-STAT3 (c) were compared between the two groups. The correlation between IL-10 and STAT3 protein levels was analyzed in all of the women (n=40) (d). Representative images of STAT3 and p-STAT3 staining in the endometria of women with adenomyosis and normal controls are shown (e). The integrated optical density (IOD) levels of STAT3 (f) and p-STAT3 (g) were compared between the two groups (n=3). The data are plotted as the mean ± SEM. *∗*P<0.05; *∗∗*P<0.01; *∗∗∗*P<0.001.

As IL-10 induced HOXA10 expression through phosphorylation of STAT3, we further detected p-STAT3 and STAT3 protein expression in the endometria of women with adenomyosis and normal controls. As shown in Figure 4, no difference in total endometrial STAT3 protein levels was found between women with adenomyosis and normal controls (Figures 4(a) and 4(b), P >0.05). However, the endometrial p(Y705)-STAT3 expression level was decreased by 50% in women with adenomyosis compared with that in normal controls (Figures 4(a) and 4(c), P<0.001), and the protein levels of p-STAT3 and IL-10 were positively correlated (Figure 4(d), r = 0.712, P < 0.01). Furthermore, the positive staining for p(Y705)-STAT3 observed in both the glandular epithelial and stromal compartments was decreased in the adenomyosis samples compared with that in normal samples, as determined by immunohistochemistry.

## 4. Discussion

Adenomyosis, which manifests as endometrial tissue within the uterine myometrium, is an endometriosis-like disease with compromised reproductive outcomes [2]. However, the exact mechanisms by which adenomyosis affects embryo implantation are unclear. Implantation is controlled by a complex and sophisticated interaction between the embryo and endometrium, which is achieved only during a very short period in the midsecretory phase. Inadequate endometrial receptivity or impaired decidualization is known to be a major limiting factor in impaired implantation [26]. In this study, we demonstrated reduced IL-10, p-STAT3, and HOXA10 expression in the endometrium of adenomyosis patients, which might lead to impaired embryo implantation.

The interleukin family plays multiple roles in embryo implantation. Abnormal expression of IL-6 has been reported in the midsecretory-phase endometrium of patients with recurrent abortions compared with that of healthy women [27]. Reduced implantation sites and fertility have been observed in IL-6-deficient mice [28]. In addition, leukemia inhibitory factor (LIF), a member of the IL-6-type cytokine family, is widely regarded as an endometrial receptivity marker that plays significant roles in both the adhesive and invasive phases of implantation [29, 30]. Furthermore, IL-11 is reported to be critical in the decidualization of stromal cells [31]. Mice lacking IL-11R*α* exhibit a fertility defect because of defective decidualization [32]. Uterine IL-10 has been demonstrated to have a dichotomous effect on human leukocyte antigen expression in trophoblast cells [20], while its function in embryo implantation is unknown. Our study demonstrated that IL-10 induces STAT3 phosphorylation, leading to increases in HOXA10 expression and the spheroid adhesion rate in vitro. Moreover, cryptotanshinone, a specific inhibitor of p-STAT3, can reverse the IL-10-induced increase in embryo adhesion.

A previous study revealed a higher staining intensity for IL-10 in both the eutopic and ectopic endometrium of women with adenomyosis than in normal controls [22]. That research focused on the pathogenesis and pathophysiology of adenomyosis, whereas our study focused on the potential underlying mechanism impairing endometrial receptivity due to adenomyosis. In this study, samples of endometrial tissues were obtained from sterile women who were diagnosed with adenomyosis. At the same time, we excluded patients with endometrial and ovarian diseases to confirm that adenomyosis was the main reason for infertility in this group. Differences in study designs, study power, and the choice of samples may explain the observed discrepancies. Another study showed that IL-10 expression is lower in endometrium secretions from women with adenomyosis than in normal controls. In this previous study, samples of endometrial tissues were obtained from patients undergoing IVF treatment during the window of implantation, which is consistent with our research [21]. Our Immunohistochemistry results revealed that IL-10 expression was significantly lower in women with adenomyosis than in normal controls. These consistent findings were observed for HOXA10 protein expression. HOXA10 is also critical for decidualization in targeted gene deletion experiments [33]. The abnormal expression of IL-10 and HOXA10 in the stroma might impair the decidualization of endometrial stromal cells in adenomyosis patients, which would result in implantation failure. Therefore, the exact functions of IL-10 and HOXA10 during decidualization in women with adenomyosis should be further assessed.

The phosphorylation of STAT3 might be a critical mediator of IL-10-induced HOXA10 expression [34, 35]. Therefore, we further detected p-STAT3 expression in endometria from women both with and without adenomyosis. The p-STAT3 level was lower in the endometria of the women with adenomyosis. We speculate that dysregulation of the IL-10/p-STAT3/HOXA10 signaling pathway results in embryo implantation failure in women with adenomyosis. The STAT3 pathway is a common signaling cascade involved in human embryo adhesion and endometrial decidualization [36–38]. IL-10 was considered to be an activator of STAT3 in the human endometrium in our research, which promoted embryo adhesion in vitro in a STAT3-dependent manner. Hence, we propose that IL-10 may be a potential endometrial receptivity marker in patients with adenomyosis. In addition, increases in adenomyosis incidence and severity are evident in tamoxifen-treated mice with increasing age, which provides a potential model for understanding the mechanism of adenomyosis [39]. Therefore, the general function of IL-10/STAT3/HOXA10 signaling in embryo implantation and the potential regulatory mechanism between the STAT3 signaling pathway and HOXA10 require further investigation in model animals and humans.

## 5. Conclusions

Both IL-10 and HOXA10 levels in the endometrium are significantly reduced in women with adenomyosis compared with those in control women. The phosphorylation of STAT3 has been proven to be a critical mediator between IL-10 and HOXA10, which may play critical roles in embryo implantation.

## Figures and Tables

**Table 1:** Demographic details of the participants in this study.

	Adenomyosis Group (n=23)	Control Group (n=23)	P
Age (years)	31.2±4.1	30.0±3.5	>0.05
Infertility time (year)	4.3±2.9	3.9±3.5	>0.05
bFSH (mIU/mL)	8.0±2.1	8.5±2.5	>0.05
AFC (n)	13.7±5.6	13.9±4.8	>0.05
Body mass index (kg/m^2^)	22.6±3.1	22.3±2.6	>0.05

The data are presented as the mean ± SD unless otherwise indicated. AFC, antral follicle count; bFSH, basal follicle stimulating hormone.

## Data Availability

The datasets used and analyzed during the current study are available from the corresponding author upon reasonable request.

## Abbreviations

IVF: In vitro fertilization
ICSI: Intracytoplasmic sperm injection
IL10: Interleukin-10
HOXA10: Homeobox A10
STAT3: Signal transducers and activators of transcription 3
IOD: Integrated optical density.
